# Structure-Based Virtual Screening and Discovery of New PPARδ/γ Dual Agonist and PPARδ and γ Agonists

**DOI:** 10.1371/journal.pone.0118790

**Published:** 2015-03-13

**Authors:** Vinicius G. Maltarollo, Marie Togashi, Alessandro S. Nascimento, Kathia M. Honorio

**Affiliations:** 1 Centro de Ciências Naturais e Humanas, Universidade Federal do ABC, Santo André, São Paulo, Brasil; 2 Faculdade de Ciências Farmacêuticas, Universidade de São Paulo, São Paulo, São Paulo, Brasil; 3 Faculdade de Ciências da Saúde, Universidade de Brasília, Brasília, Distrito Federal, Brasil; 4 Instituto de Física de São Carlos, Universidade de São Paulo, São Carlos, São Paulo, Brasil; 5 Escola de Artes Ciências e Humanidades, Universidade de São Paulo, São Paulo, São Paulo, Brasil; University of Edinburgh, UNITED KINGDOM

## Abstract

Peroxisome proliferator-activated receptors (PPARs) are involved in the control of carbohydrate and lipid metabolism and are considered important targets to treat diabetes mellitus and metabolic syndrome. The available PPAR ligands have several side effects leading to health risks justifying the search for new bioactive ligands to activate the PPAR subtypes, in special PPARδ, the less studied PPAR isoform. Here, we used a structure-based virtual screening protocol in order to find out new PPAR ligands. From a lead-like subset of purchasable compounds, we identified 5 compounds with potential PPAR affinity and, from preliminary *in vitro* assays, 4 of them showed promising biological activity. Therefore, from our *in silico* and *in vitro* protocols, new PPAR ligands are potential candidates to treat metabolic diseases.

## Introduction

Peroxisome proliferator-activated receptors (PPARs) constitute a subfamily of nuclear receptors involved in the transcription of genes related to the cellular proliferation and differentiation, immune responses and metabolism of carbohydrates and lipids [1–8]. From the pathological point of view, these receptors are related to metabolic diseases (mainly, type 2 diabetes mellitus, metabolic syndrome and dyslipidemia) [9–11], inflammatory process [12,13], neurodegeneration [14] and some kinds of cancer [15–17].

The treatment of type 2 diabetes mellitus (T2DM) and metabolic syndrome (MS) brings an important focus to the development of new PPAR agonists. There are at least two classes of pharmacological agents targeting the PPARs. The fibrates are known as PPARα ligands used for the control of hypercholesterolemia, while the thiazolidinediones (TZDs) such as rosiglitazone and pioglitazone are PPARγ full agonists used as insulin sensitizers in T2DM therapy. Despite the clear efficacy to restore blood glucose levels, rosiglitazone was reported to cause important side effects, such as fluid retention, weight gain and increase in the chance of a cardiovascular event [18]. These effects leaded some regulatory agencies around the world to restrict or suspend the use of rosiglitazone [19]. Interestingly, pioglitazone was shown to be a safer drug than rosiglitazone, raising two interesting considerations about the interactions between PPARγ and its agonists. As noted by Bruning and coworkers [18], small changes in the binding mode, as observed between rosiglitazone and pioglitazone, can lead to important changes in the pharmacological profile, including the side effects.

There is another class of PPAR agonists, fibrates, responsible for the activation of PPARα that is well known to result in a decrease in the triglyceride levels and increase in HDL levels (considered important factors for the establishment of T2DM) [20]. On the other hand, PPARδ (the less studied receptor among the PPARs) was shown to be involved in anti-obesity effects [21] and anti-inflammatory processes as arthritis, eczema and psoriasis [22,23] reinforcing the potential beneficial effects of this receptor in the treatment of chronic and metabolic diseases. Most PPAR dual- and pan-agonists developed to date were discontinued during the clinical trials due to harmful effects observed in these assays, making unclear whether these agonists can actually exert *in vivo* beneficial effects [20]. Researches on these topics are very important have the main purpose of highlighting how relevant it is the development of new PPARδ agonists that could be used as chemical probes for a better understanding on the molecular mechanisms involved in the PPARδ activation.

Here, a virtual screening (VS) of a large chemical library, the ZINC database [24], based on the crystallographic structure of PPARδ receptor was used as a tool to identify new PPAR ligand candidates. PPAR agonists can be classified as full agonists (related to the stabilization of α-helix 12 [H12] by the interaction with polar residues and, this way, providing structural conditions to the recruitment of cofactors at the AF-2 region) and partial agonists (related to a suboptimal stabilization of H12 and also called H12 independent mechanism) [18]. Since the main commercial PPAR agonists (glitazones and fibrates) and the reference PPARδ agonist (GW501516) are full agonists [25,26], our VS protocol was carried out aiming to select the compounds by analyzing the interactions that characterize a full agonist. After several analyses of the main ligand-receptor interactions, five compounds were selected for preliminary biological assays and four novel ligands were identified as agonists acting on PPARδ/γ and PPARγ. These ligands are promising candidates to treat metabolic disorders, such as diabetes and metabolic syndrome.

## Material and Methods

### Molecular Docking

The ‘clean-leads’ subset of purchasable compounds library was chosen for virtual screening as available in ZINC (version of January-2010) [24]. This subset includes purchasable compounds with molecular weight between 250 and 350 Da, n-octanol/water partition coefficient values between 2.5–3.5 and number of rotatable bonds between 5 and 7 and this subset also excludes toxic compounds with aldehydes and thiol groups.

For the docking simulations, the crystal structure 3GZ9 containing PPARδ LBD was chosen [27] and prepared in three steps. First, all non-amino acid atoms (waters, ions, ligands and others) were removed from the structure. Then, the missing hydrogen atoms were added and, finally, the protonation state of the residues in the active site was automatically defined based on the local interactions. All water molecules were removed from the active site, since there are no structural water molecules mediating the main interactions responsible for the PPARδ activation from the selected crystallographic structure. The preparation of the receptor structure was performed with UCSF Chimera [28] software. Afterwards, the active site region was defined based on the coordinates of the crystallographic ligand. Finally, the semi-flexible docking simulations were performed employing DOCK 3.5.54 [29–35] software. The ligand conformers were obtained from ZINC database [24].

50 molecules selected after several analyses of the docking results were used for redocking using Surflex-docking [36] and GOLD 3.1 [37–39] with their default parameters. The Surflex-docking software employs the incremental search to generate docking poses, whereas GOLD 3.1 software uses a genetic algorithm (GA) to generate the poses of ligands. Both Surflex-docking and GOLD programs generated 50 poses for each ligand and the top 5 ranked poses were analyzed. The ranking of the docked poses was performed using CScore and GOLD-Score, respectively. All the generated poses were analyzed with a rigorous visual inspection regarding the main molecular interactions between some amino acid residues in the active site and the studied ligands. In this analysis, the polar interactions with Thr-289, His-323, His-449 and Tyr-473 and also stereochemical complementarities were inspected.

The main purpose of using three different docking programs to perform a consensus analysis is avoiding the pitfalls of each method. DOCK 3.5.54 and GOLD 3.1 programs use force-field based scoring function [34,39] while Surflex-docking uses a consensus scoring function (Chem-Score as empirical scoring function, D-score and G-score as force-field based scoring function and PMF-score as knowledge-based scoring function) [40]. These programs also have important differences in the generation of ligand conformers. The definition of active site (rectangular box, sphere centered or amino acid residues) and the flexibility degree of ligand bonds/angles can also affect the success rate in the molecular docking because it interferes in the search space of the docking algorithm. Finally, taking into account that the training set used to calibrate each docking engine is different from each other, it is expected that a docking program works better on a certain system than others [41]. Several studies indicate that it is too difficult to compare the efficiency of docking programs due to several factors such as the limitation of evaluation metrics (e. g. root mean squared deviation [RMSD] value could indicate an acceptable pose but the ligand orientation is wrong) [41–43]. Therefore, a consensus between the ranked poses and a careful visual inspection of the results obtained by the three different algorithms could improve the final results [44,45].

### Transluciferase Assays

HeLa cells were cotransfected [46,47] by electroporation with expression vectors pcDNA3-PPARδ or pcDNA3-PPARγ, report vector pGL3-PPARE and pRL. In addition, the cells were treated for 20h in the presence of the studied ligands. Bezafibrate was employed as positive control to PPARδ and rosiglitazone was employed as positive control to PPARγ. DMSO was employed as vehicle to compounds 1–4 and the DMSO:EtOH (2:1 v/v) mixture was employed as vehicle for compound 5. The PPAR activation measurements were estimated by Luciferase Assay System (Promega) and were determined by the standard deviation of triplicate measurements. Unfortunately, the transactivation assays with PPARα were not experimentally accessible for us.

### Molecular Dynamics (MD) Simulations

Before the MD simulations, we generated the initial conformation of the compound 1 in the PPARδ and γ binding sites and compound 2 at PPARγ binding site using DOCK 3.4 software with the same VS protocol. We used the 3GZ9 [27] structure as PPARδ model and 1ZGY [48] as PPARγ model.

All MD parameters were equally set to the four generated models. The MD simulations were performed employing the GROMACS v.4.5.4 software [49,50] in an Intel Xeon processor with 8GB RAM, running the CentOS5.5 Linux operating system. The explicit water molecules were defined employing Simple Point Charge (SPC) model [51]. Protonation states of some amino acid residues were set according to pH 7.0 and counter ions were added to neutralize the system. The protonation states of histidine residues were set as default (epsilon form), because the initial protonation was tested only with docking protocol and for PPARδ. Gromos force field [52] was chosen to perform the MD simulations. The ligand topologies were generated employing PRODRG2 Server [53], which also uses Gromos force field to parameterize charges and protonation states. The MD simulations were performed at constant temperature and pressure in a periodic truncated octahedral box, with a minimum distance between box edges and any protein atom equal to 2.0 nm.

Initially, an energy minimization using a steepest descent algorithm was performed. Then to equilibrate the system, 200 ps of MD were performed at 298 K with positional restraints applied to the backbone atoms using LINCS algorithm. Finally, an unrestrained MD was performed at 298 K during 5 ns of simulation to assess the stability of the structures. During the simulations, temperature and pressure (1.0 bar) were maintained by the coupling to an external heat and an isotropic pressure bath. Finally, we generated all MD figures employing PyMOL 0.99c [54] software.

## Results and Discussion

### Structure-based Virtual Screening

From the 21 crystallographic structures of PPARδ [27,55–68] found in Protein Data Bank (PDB), we selected the structure with PDB code 3GZ9 [27] due to its crystallographic quality (the lowest resolution value equals to 2Å, R-value and R-free values equal to 0.192 and 0.254, respectively) and the similarity of its crystallographic ligand to the compound dataset employed in the enrichment-based model calibration stage. 51 PPARδ ligands synthesized and tested by Wickens *et al*. [69] were employed as active compounds, while a subset of PPAR decoys (~ 3600 compounds) from Directory of Useful Decoys (DUD) [70] was employed as inactive compounds in the enrichment-based calibration. In this step, we performed the docking analysis of all actives ligands and decoys by varying several internal parameters related to ligand orientation (distance tolerance; the number and histogram parameters of ligand-receptor spheres matching; the minimum and maximum number of ligand atoms to consider it as docked; distance and degrees of molecule initial translation and rotation, respectively) and the use of “chemical matching” function of DOCK 3.5.54. The chemical matching function consists in creating spheres related to a specific compatible chemical group (H-bond donor or acceptor, charged groups and hydrophobic group) into the active site and trying to fit the correspondent groups of the ligand in the created spheres. In other words, chemical matching is a function with the idea of pharmacophore matching. The default settings of DOCK 3.5.54 without chemical matching function showed the best performance to distinguish the active compounds to decoys identifying all active compounds at 5% of all screened dataset.

**Fig 1:**
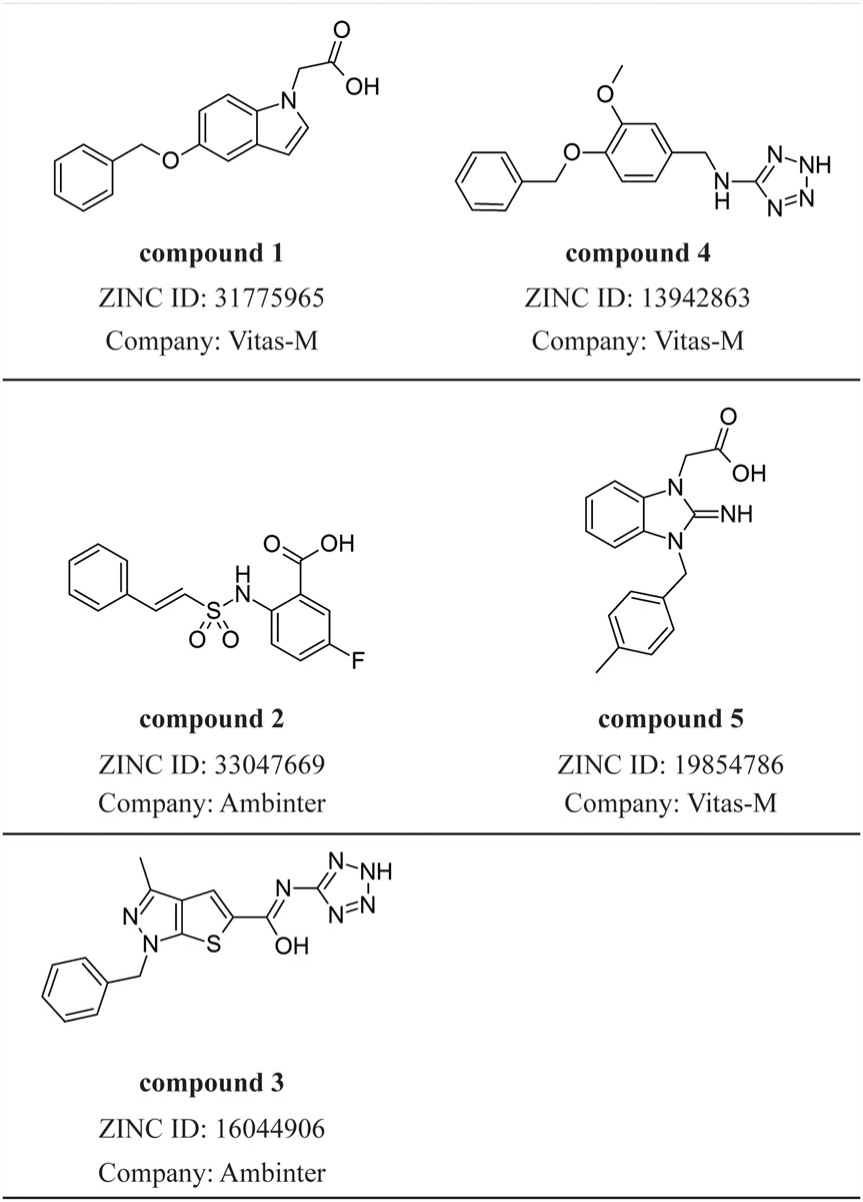
Compounds selected by VS protocol.

740,000 compounds were docked in the active site of PPARδ using the program UCSF DOCK 3.5.54 [29–35] and, then they were sorted from the calculated binding energy. Fifty compounds (approximately 0.007% of the initial set) were selected in a visual inspection from the docking results (A Table in S1 File). The fifty selected compounds were then redocked in the PPARδ active site using the Surflex-Dock [36], as implemented in Sybyl 8.1 package [71], and Gold 3.1 [37–39] with their default parameters. According to the agreement among DOCK, Surflex and Gold poses and binding energies, five compounds were selected for the biological evaluation. Fig. 1 shows the chemical structure of the selected ligands.

### Biological Assays

Initially, luciferase transactivation assays were carried out to verify the activity of the chosen compounds against PPARδ and γ in comparison to negative (vehicle) and positive (bezafibrate, 100 μM for PPARδ; rosiglitazone, 100 μM for PPARγ) controls (A Fig. in S1 File). For an initial screening, the compounds were tested in different concentrations, according to the ligand solubility in DMSO. The most promising results were observed for the compounds 1 (300 μM) and 5 (10 μM) which activated PPARδ by 5.9 and 6.4 fold than vehicle and, surprisingly, by compound 2 (3 μM) which activated only PPARγ by 6.9 fold than vehicle. The compound 1 also showed PPARγ activity (6.8 fold of activation) at the same concentration of the positive control and compound 4 showed a small activity against PPARδ and γ (2.5 and 2.8 fold in comparison to vehicle, respectively) at 10 μM and 100 μM, respectively.

After the first experimental assays, two compounds were selected for a deeper examination. Compound 1 was chosen due to its dual agonistic profile on PPARδ and γ. On the other hand, compound 2 was found as selective PPARγ agonist. Dose-response curves were generated from the luciferase transcriptional activation for the compound 1 using PPAR β/δ and PPARγ (B Fig. in S1 File). EC_50_ of 134.2 μM and 18.1 μM were found for PPARδ and PPARγ, respectively, confirming that the compound 1 is more potent as PPARγ agonist than as PPARδ agonist. Compound 2 showed an EC_50_ value of 190.8 μM. Dose-response curves could not be generated for the compound 5, since higher doses resulted in a marked decrease in the cellular activation. The compounds 1 and 2 showed EC_50_ above than 100 μM which is the limit to consider a compound as a hit. On the other hand, all 5 selected compounds are negatively charged at physiological pH due to its acid functions and, then, the low membrane permeability may influence the compounds to reach the PPAR binding sites [72]. Then, the high obtained EC_50_ values could be explained by this factor and the compounds 1 and 2, specifically, could be explored by further SAR experiments in order to generate derived compounds with high PPAR binding affinity.

### Molecular Dynamics Simulations

In order to study the binding mode of the most active compounds and their PPAR selectivity, we performed various molecular dynamics (MD) simulations. We selected the compounds 1, 2 and 5 to perform the simulations due to the significant PPAR activation in comparison to the vehicle and the positive control. The docking poses were selected as initial conformations in the MD simulations. We selected the compounds 1, 2 and 5 (the most active compounds) in complex with PPARδ and γ and we also made MD simulations for the PPAR unbound subtypes for comparison.

An equilibrium state was reached in all simulations after 4 ns, as observed by the RMSD values, available in the Supplementary Material (C Fig. in S1 File). The RMSD values for the ligands in the active sites and all H-bonds performed between the ligands and the PPAR receptors are shown in Supplementary Figs. (D and E Figs. in S1 File, respectively). From these results, we analyzed the interactions between the selected ligands (1, 2 and 5) and the main residues in the active site of all PPAR subtypes after stabilization in order to understand their behavior in the PPAR active sites, as well as their experimental selectivity. Finally, aiming to study the occupancy of the ligands into the H12 region, we analyzed the number of H-bonds between the compounds and some residues in each active complex.

### Compound 1

**Fig 2:**
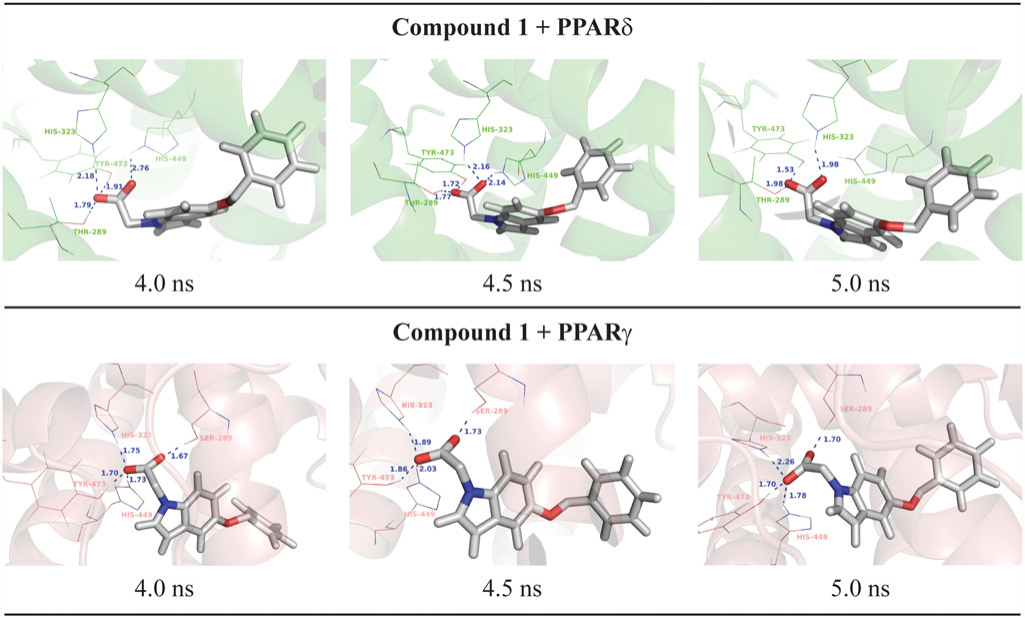
Final nanosecond snapshots of compound 1 molecular dynamic simulations in complex with PPAR subtypes. Main polar interactions between the compound 1 and the PPARδ and PPARγ during the final nanosecond of MD simulation.

**Fig 3:**
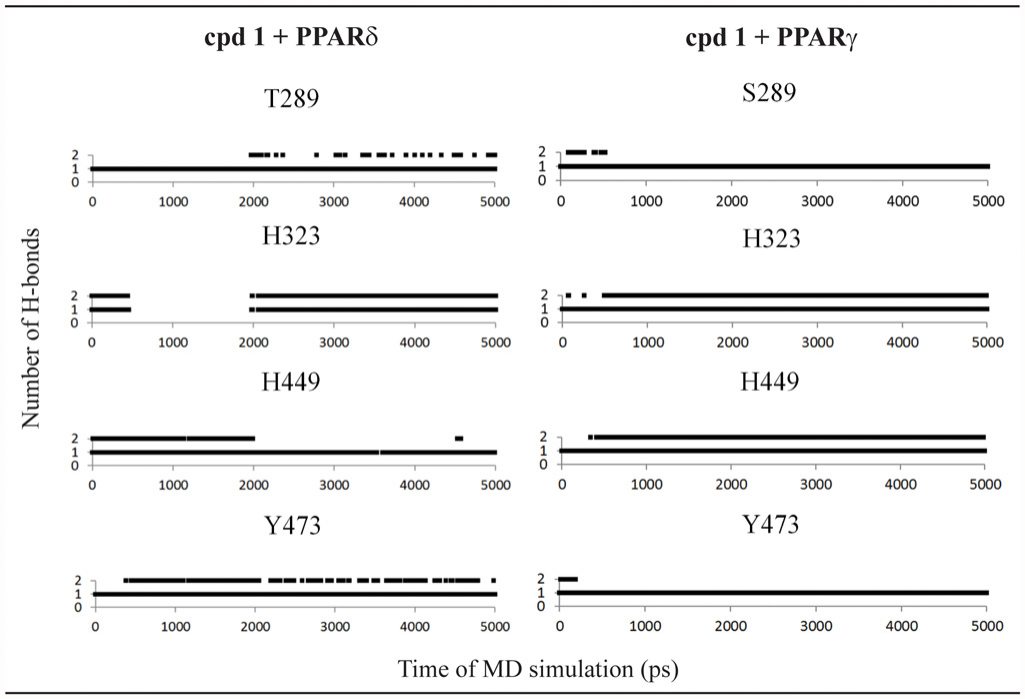
Hydrogen bonds diagram between compound 1 and polar residues of PPARδ and PPARγ. Black squares indicate the presence of H-bonds and white ones correspond to the absence of H-bonds.

For the [5-(benzyloxy)-1H-indol-1-yl]acetic acid (compound **1**, Fig. 2), H-bonds with all polar residues in the PPAR active site for all subtypes (at least one His or Tyr residues) were observed in the MD simulations (Fig. 3). These polar interactions involve H323, H449, T289 and Y473 in PPARδ; and H323, H449, Y473 and S289 in PPARγ (Fig. 2). A similar binding mode was found to PPARδ and PPARγ, a typical behavior for PPAR full agonists. An analysis of the occupancy for the hydrogen bonds (Fig. 3) shows that the interaction of the compound 1 with the tyrosine residue located in H12 is more stable for PPARδ and PPARγ [64].

### Compound 2

**Fig 4:**
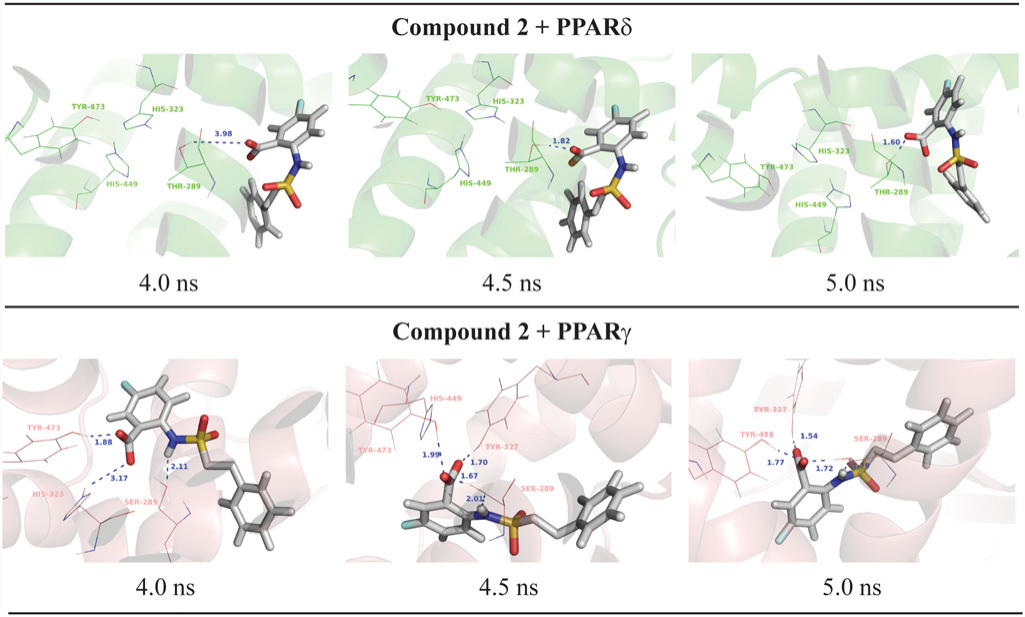
Final nanosecond snapshots of compound 2 molecular dynamic simulations in complex with PPAR subtypes. Main polar interactions between the compound 2 and the PPARδ and PPARγ during the final nanosecond of MD simulation.

**Fig 5:**
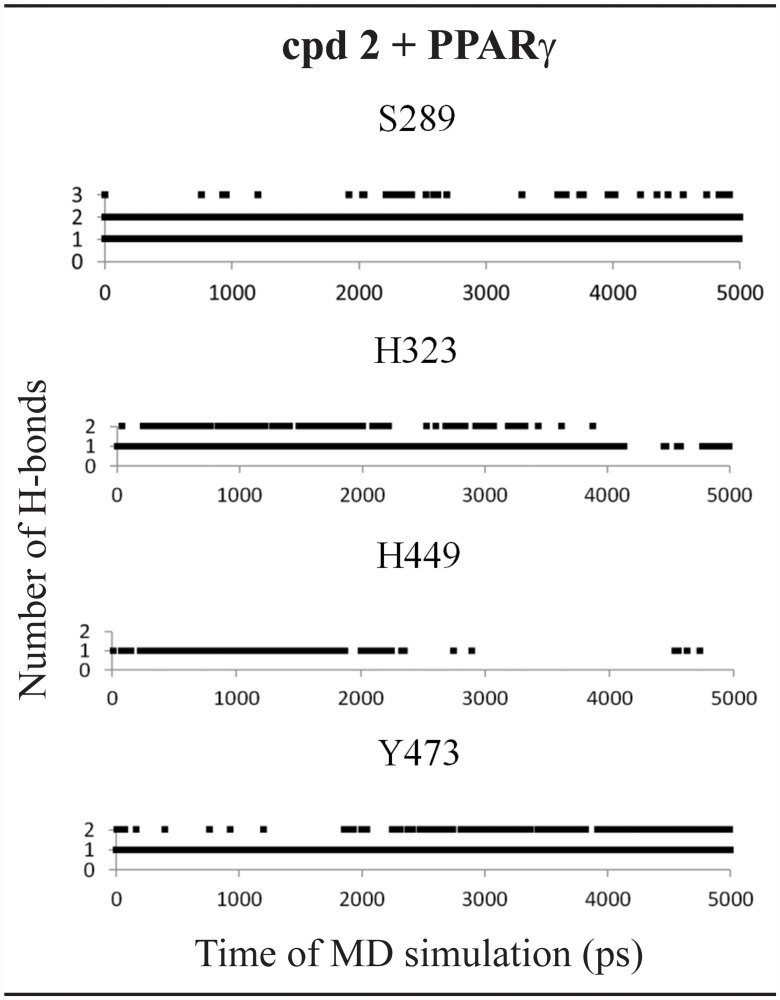
Hydrogen bonds diagram between compound 2 and polar residues of PPARγ. Black squares indicate the presence of H-bonds and white ones correspond to the absence of H-bonds.

Surprisingly, unlike docking results obtained for PPARδ, 5-chloro-2-{[(2-phenylethyl)sulfonyl]amino}benzoic acid (compound **2**) showed only activation on PPARγ. Indeed, considering the interactions of this compound in the active site, this ligand is not able to maintain the polar interactions with PPARδ (Fig. 4), explaining its PPARγ selectivity. At the PPARδ binding site, the compound 2 forms H-bonds with some residues (Thr-289 of PPARδ) and is not able to reach the polar cavity of both receptors (Fig. 5). This can also be explained due to the larger binding cavity of PPARγ than δ [6,64,73,74]. At the PPARγ active site, the compound 2 is able to perform and maintain polar interactions with Ser-289, His-323, His-449 and Tyr-473 along the 5ns of MD simulation according to Figs. 4 and 5.

### Compound 5

**Fig 6:**
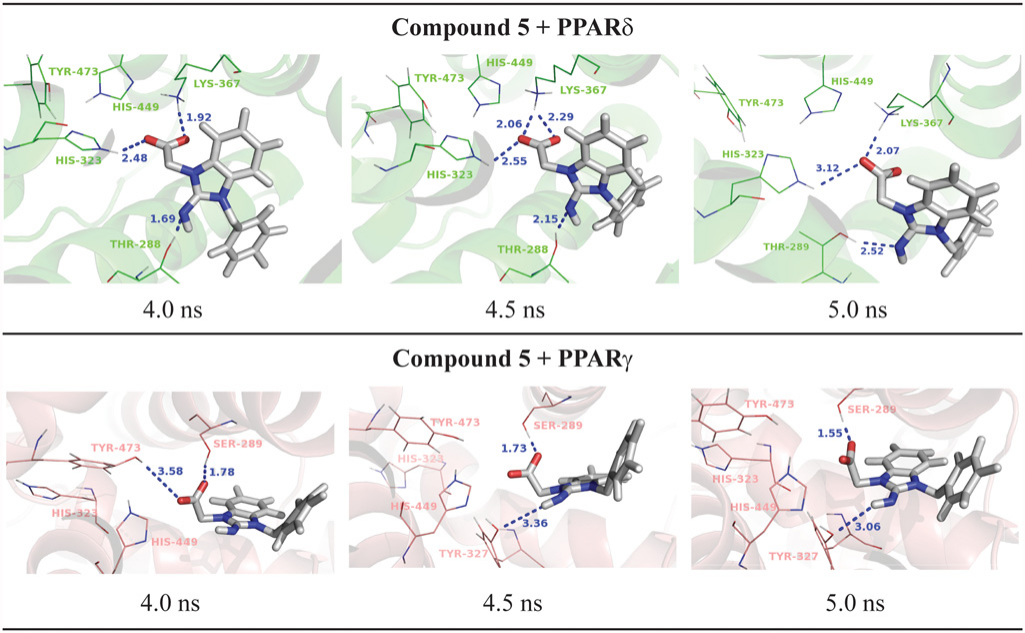
Final nanosecond snapshots of compound 5 molecular dynamic simulations in complex with PPAR subtypes. Main polar interactions between the compound 5 and the PPARδ and PPARγ during the final nanosecond of MD simulation.

Finally, the compound 5 ([2-imino-3-(4-methylbenzyl)-2,3-dihydro-1H-benzimidazol-1-yl]acetic acid) showed PPARδ activation from our preliminary experimental assays. In line with the transactivation results, the MD simulations showed only loose interactions between this ligand the PPARγ binding pocket. As shown in Fig. 6, the compound 5 directly interacts with His-323 and Lys-367 and acts as an H-bond acceptor interacting with Thr-288 of PPARδ; on the other hand, in the PPARγ active site, the compound 5 interacts only with Ser-289. The lack of strong interactions leads the ligand to move away from the active site even in short dynamics (5 ns). Therefore, one possible reason for a missing activity of the compound 5 on PPARγ is the lack of interactions with histidine or tyrosine residues as the compounds 1 and 2 perform, showing the importance of these residues in the PPARγ activation.

**Fig 7:**
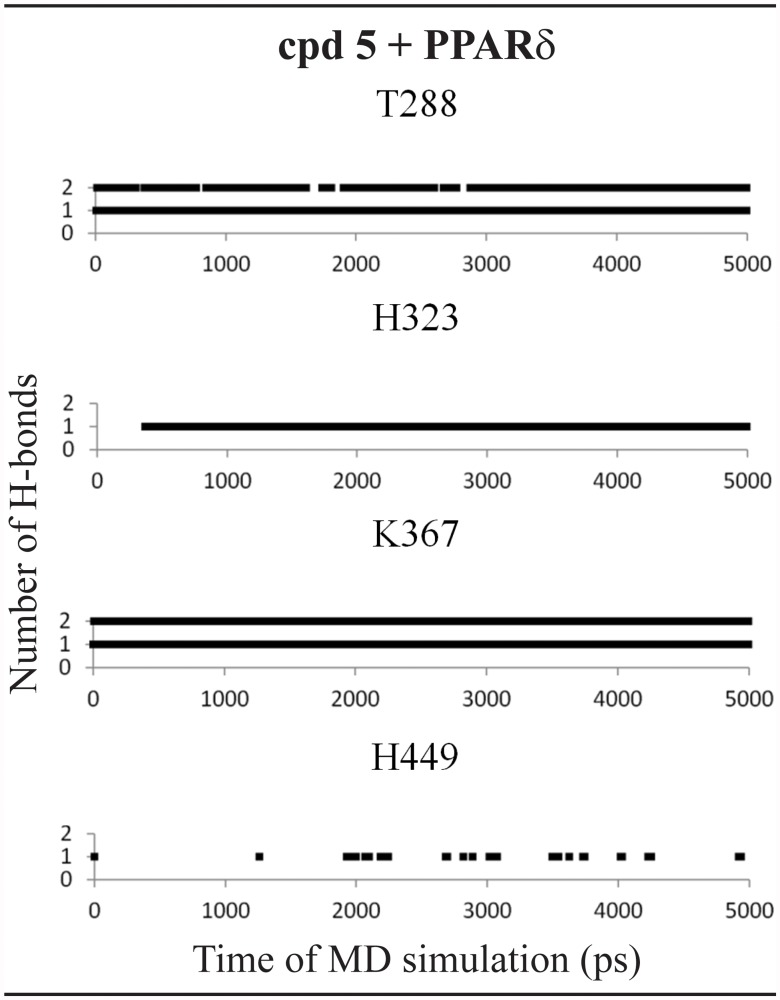
Hydrogen bonds diagram between compound 5 and polar residues of PPARδ. Black squares indicate the presence of H-bonds and white ones correspond to the absence of H-bonds.

In the PPARδ active site, this ligand forms H-bonds with two histidine residues (His-323 and His-449) during all MD simulation and two additional interactions between Thr-288 and Lys-367 (Fig. 7).

In this study, we can conclude that the compounds selected by the virtual screening were able to perform the main polar contacts with PPARδ and γ, which are a way to activate this nuclear receptor providing the conditions to the gene transcription [73,75–81]. There are several experimental and theoretical studies indicating the different behavior of PPAR ligands [82–85]. However, to the best of our knowledge, there are few studies involving PPARδ ligands, proving the importance of our study. Here, the results obtained from the MD analyses are able to explain the behavior of the active compounds at the PPAR active sites. Based on the experimental and computational analyses, obtained in this study, we can conclude that our structure-based studies were successfully carried out and a new scaffold of PPAR ligands was found out.

## Conclusion

In this study, we performed a structure-based virtual screening using a PPARδ structure aiming to find out new molecular entities with PPAR affinity. Then, the clean-leads ZINC subset was employed as ligand database and the docking analyses were performed with DOCK program. The binding energies and visual inspections were used to rank the compound library. Finally, a consensus analysis using GOLD and Surflex-Dock programs was carried out and 5 substances with potential PPAR affinity were selected. From the 5 purchased compounds, 4 of them presented potential biological activity: compounds 1 and 4 showed PPARδ/γ activity; compound 2 displayed a significant PPARγ activation and; finally, compound 5 presented as a PPARδ agonist. In addition to the new found scaffold, it is important to mention that the tetrazole group is present in the compound 4 (with low levels of activation) as well in 13 of the first 50 compounds ranked by DOCK (A Table in S1 File). Indeed, the tetrazole moiety is a well-known bioisosteric replacement for acids [86]. So, the presence of tetrazole in our findings is not surprisingly, but the experimental evidences for the activity of the compound 4 support the use of this ligand in further investigations. Therefore, the tetrazole group can act as the polar head present in the typical PPAR ligands and can be explored in future SAR studies.

## Supporting Information

S1 File Structure of the selected compounds, results from the biological assays and MD simulations. **A Fig**. Luciferase assays. Activation of PPARδ (A) and PPARγ (B) at the single concentration of the 5 selected ligands. **B Fig**. EC_50_ values of the compounds 1 and 2. **C Fig**. RMSD values for the protein backbone during the MD simulation. **D Fig**. RMSD values for the ligand atoms during the MD simulation. **E Fig**. Number of H-bonds between the selected ligands and the protein atoms during the MD simulation. **A Table**. 50 compounds selected by DOCK. These compounds were employed in the redocking analyses using GOLD and Surflex programs.
(ZIP)
