# Supplementary material for: Structure-Based Virtual Screening and Discovery of New PPARδ/γ Dual Agonist and PPARδ and γ Agonists
Source: PLoS One. 2015 Mar 13;10(3):e0118790. doi: 10.1371/journal.pone.0118790 (PMC4358979; doi:10.1371/journal.pone.0118790)
Supplement: S1 File — A Fig. Luciferase assays. Activation of PPARδ (A) and PPARγ (B) at the single concentration of the 5 selected ligands. B Fig. EC50 values of the compounds 1 and 2. C Fig. RMSD values for the protein backbone during the MD simulation. D Fig. RMSD values for the ligand atoms during the MD simulation. E Fig. Number of H-bonds between the selected ligands and the protein atoms during the MD simulation. A Table. 50 compounds selected by DOCK. These compounds were employed in the redocking analyses using GOLD and Surflex programs. (ZIP) [file pone.0118790.s001.zip › S1_File/A_Table.pdf]

Table S1

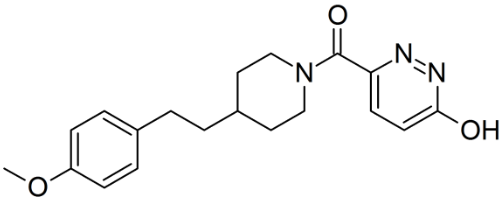	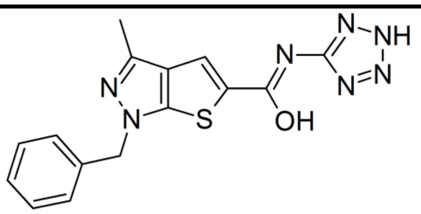	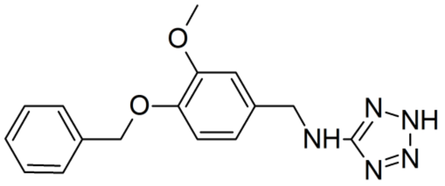
**1 -** ZINC32869794	**2 -** ZINC16044906	**3 -** ZINC13942863
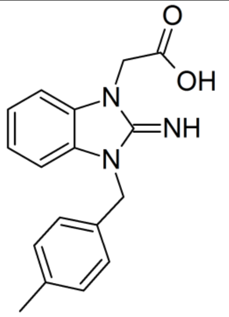	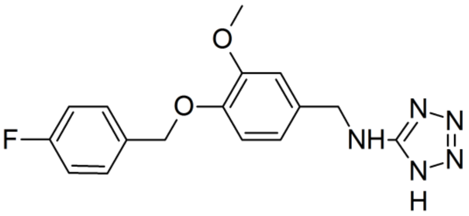	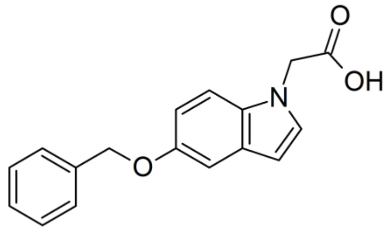
**4 -** ZINC19854786	**5 -** ZINC19871610	**6 -** ZINC31775965
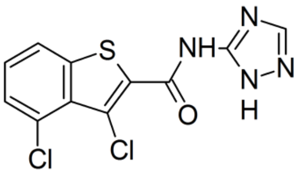	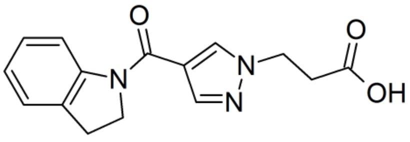	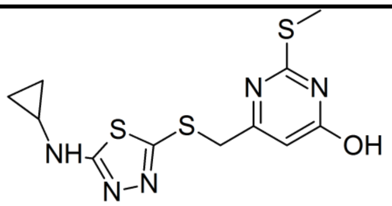
**7 -** ZINC26258041	**8 -** ZINC20325257	**9 -** ZINC08937015
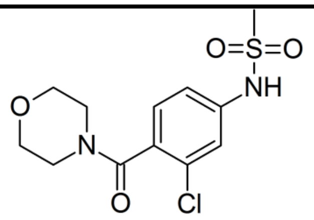	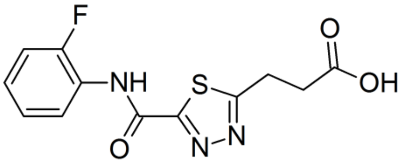	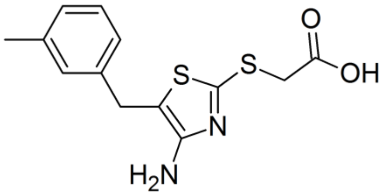
**10 -** ZINC20107347	**11 -** ZINC34927193	**12 -** ZINC16789810
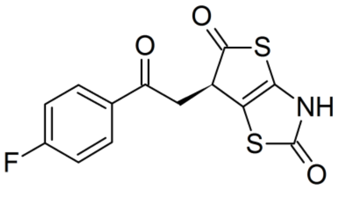	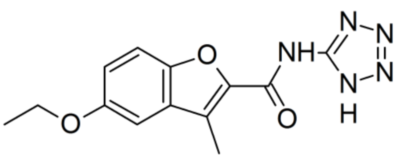	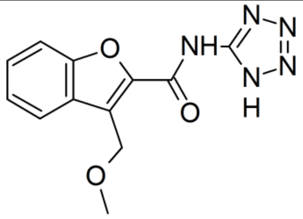
**13 -** ZINC07699133	**14 -** ZINC12564270	**15 -** ZINC23143132
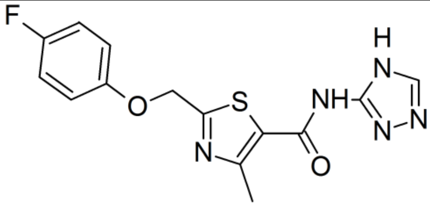	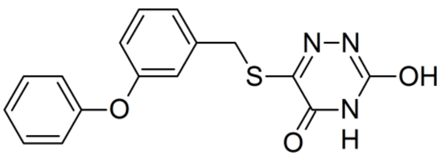	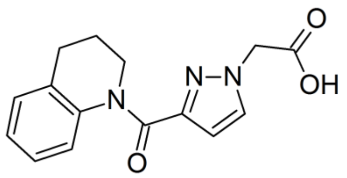
**16 -** ZINC23141006	**17 -** ZINC05094971	**18 -** ZINC20316206

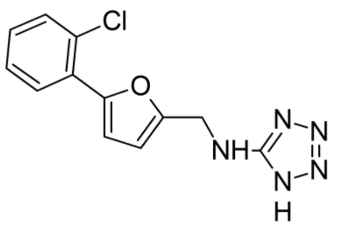<br>**19 -** ZINC20877395	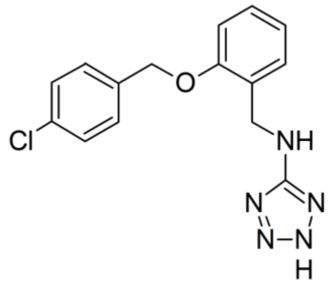<br>**20 -** ZINC16449798	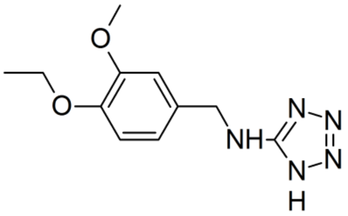<br>**21 -** ZINC13941904
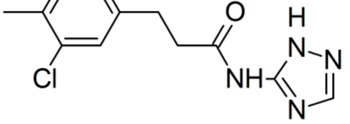<br>**22 -** ZINC23141257	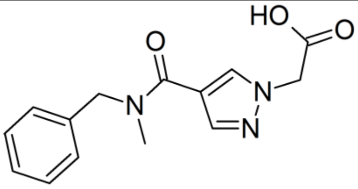<br>**23 -** ZINC20323468	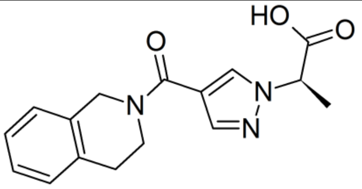<br>**24 -** ZINC20324068
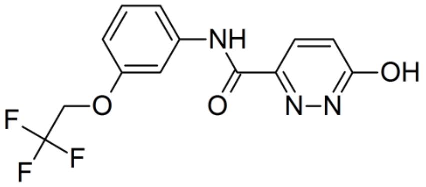<br>**25 -** ZINC31608929	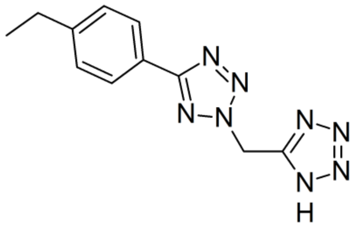<br>**26 -** ZINC12715316	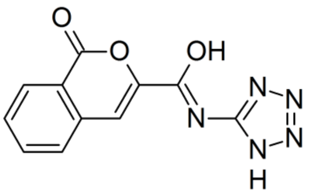<br>**27 -** ZINC19898412
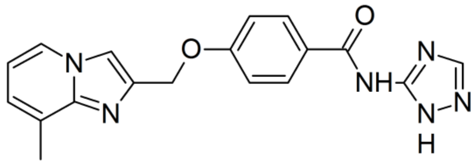<br>**28 -** ZINC23141050	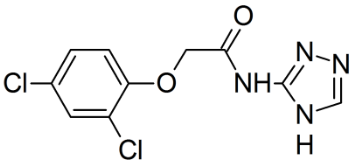<br>**29 -**ZINC06091953	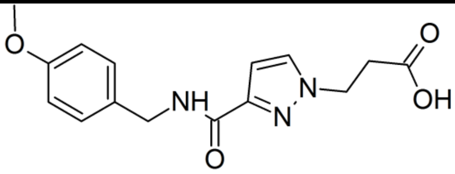<br>**30 -** ZINC20318016
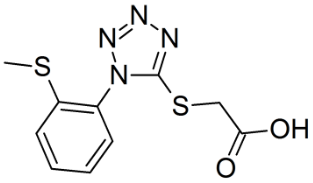<br>**31 -** ZINC08496937	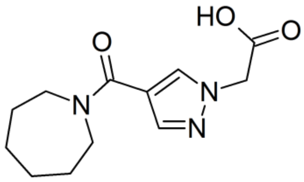<br>**32 -** ZINC20322897	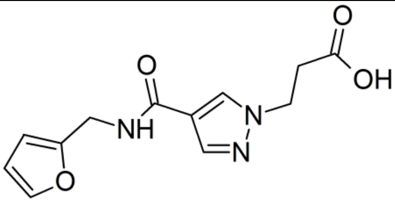<br>**33 -** ZINC20325110
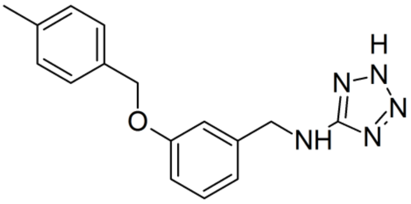<br>**34 -** ZINC04999773	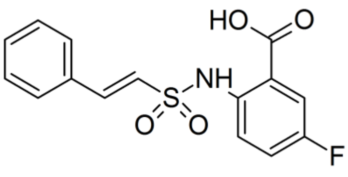<br>**35 -** ZINC33047669	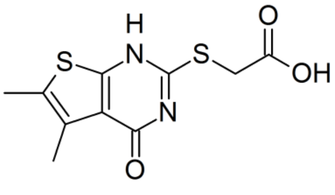<br>**36 -** ZINC19681634
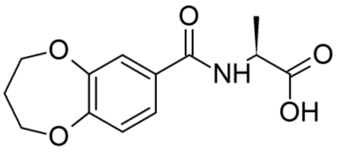<br>**37 -** ZINC28313712	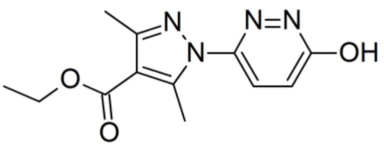<br>**38 -** ZINC13394892	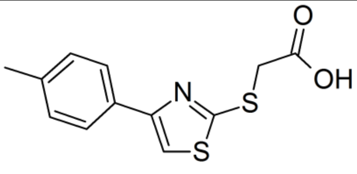<br>**39 -** ZINC32719098

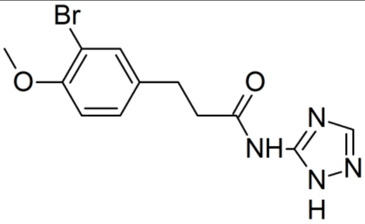

40 - ZINC23140688

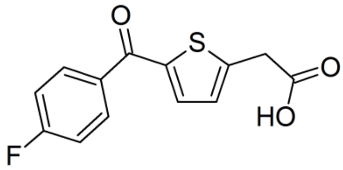

41 - ZINC05344425

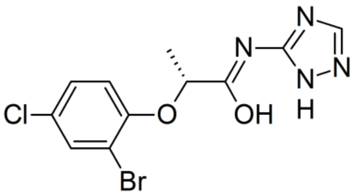

42 - ZINC23140759

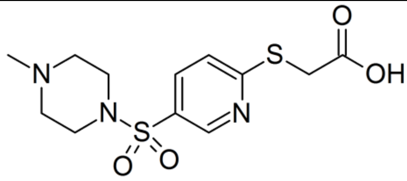

43 - ZINC34889845

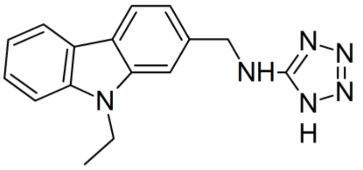

44 - ZINC19872300

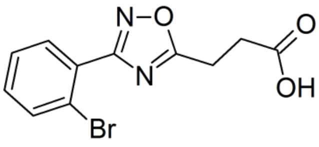

45 - ZINC17087020

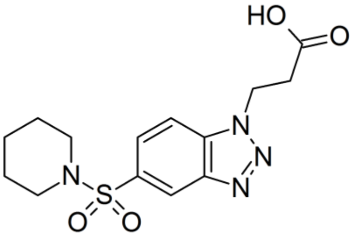

46 - ZINC15775070

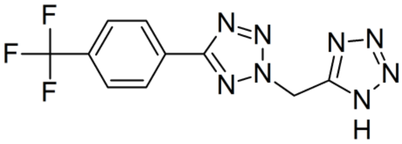

47 - ZINC04753766

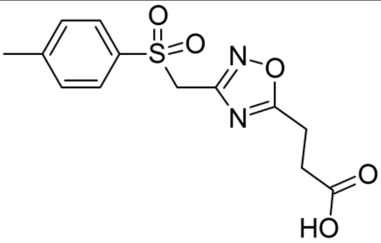

48 - ZINC21779134

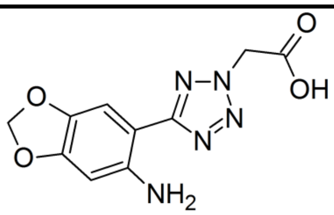

49 - ZINC20120476

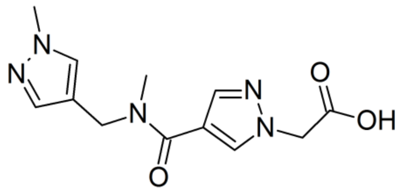

50 - ZINC20323158
