# Supplementary figures and images for: Structure-Based Virtual Screening and Discovery of New PPARδ/γ Dual Agonist and PPARδ and γ Agonists
Source: PLoS One. 2015 Mar 13;10(3):e0118790. doi: 10.1371/journal.pone.0118790 (PMC4358979; doi:10.1371/journal.pone.0118790)

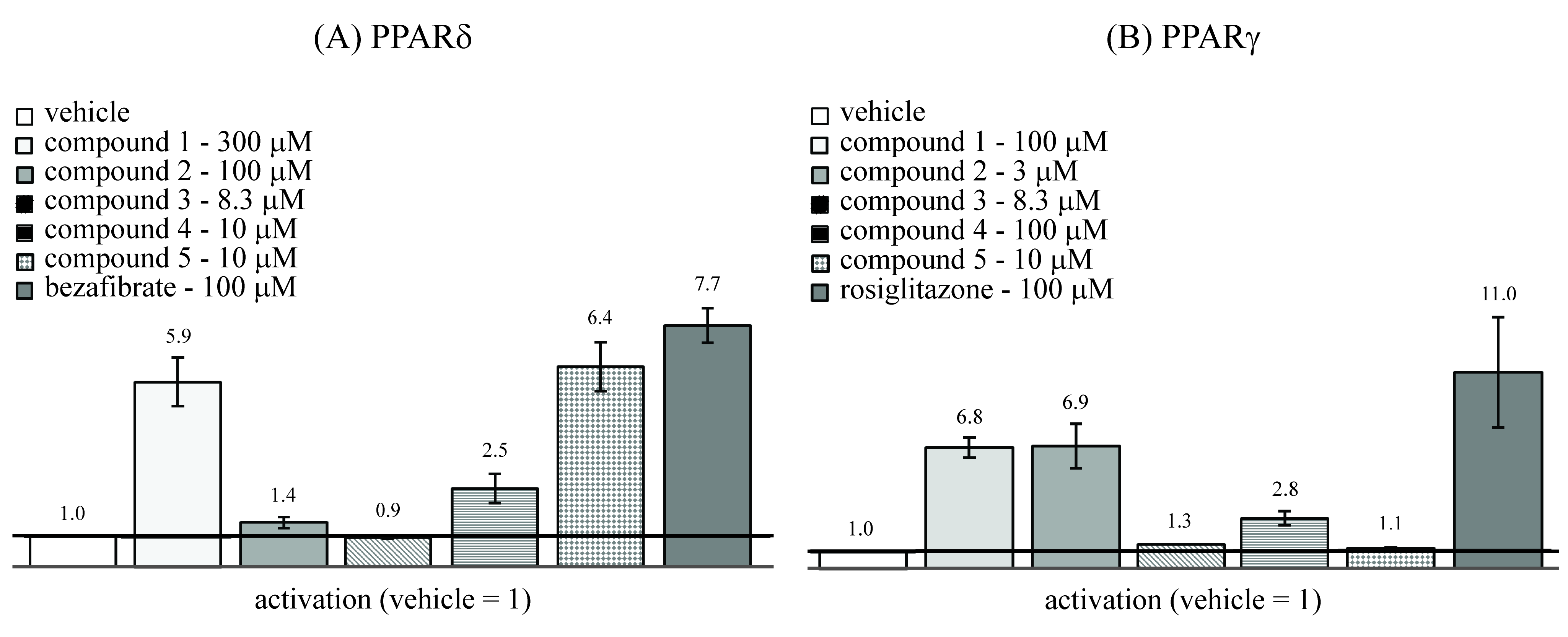

Supplement: S1 File — A Fig. Luciferase assays. Activation of PPARδ (A) and PPARγ (B) at the single concentration of the 5 selected ligands. B Fig. EC50 values of the compounds 1 and 2. C Fig. RMSD values for the protein backbone during the MD simulation. D Fig. RMSD values for the ligand atoms during the MD simulation. E Fig. Number of H-bonds between the selected ligands and the protein atoms during the MD simulation. A Table. 50 compounds selected by DOCK. These compounds were employed in the redocking analyses using GOLD and Surflex programs. (ZIP) [file pone.0118790.s001.zip › S1_File/A_Fig.tif]

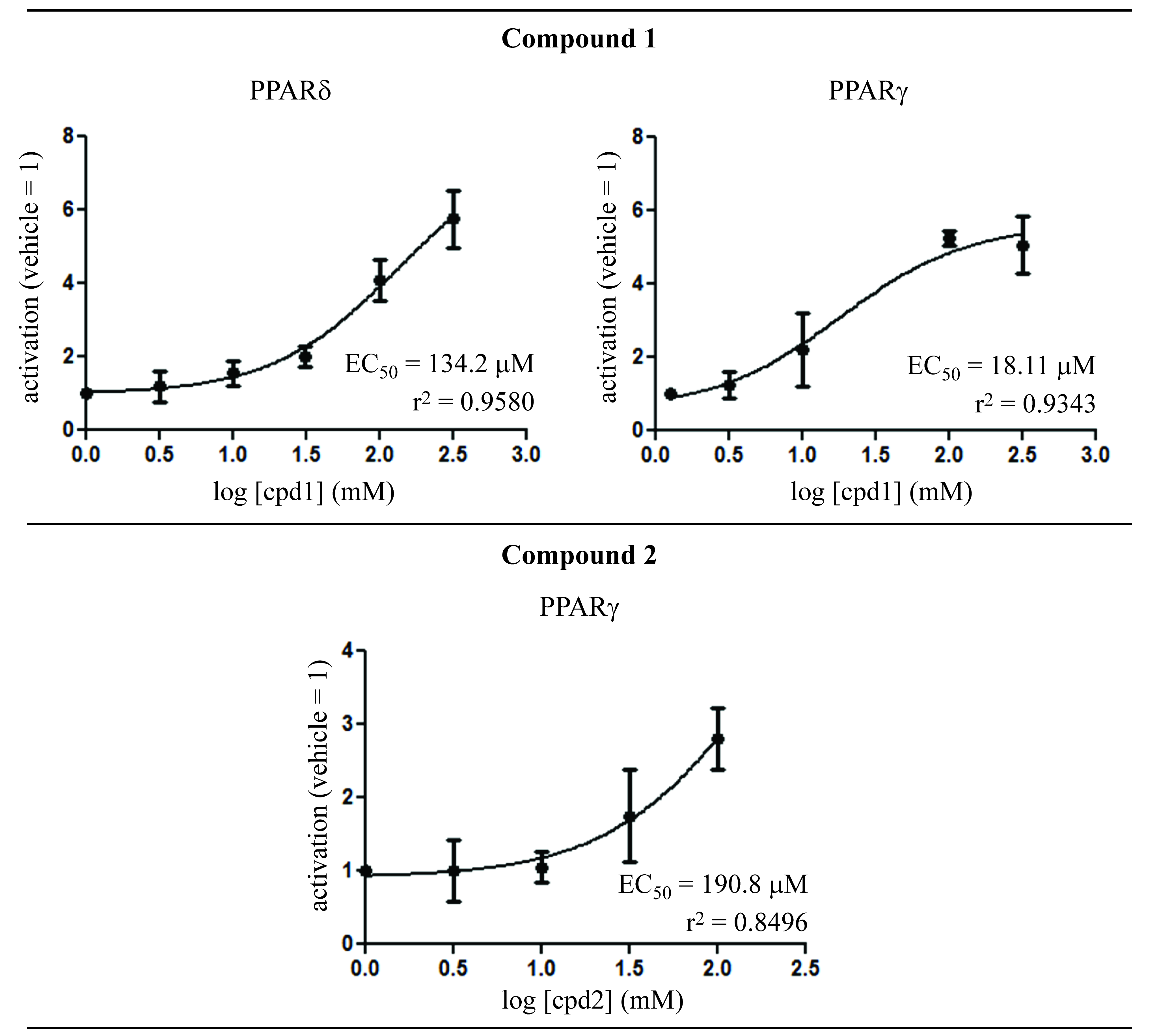

Supplement: S1 File — A Fig. Luciferase assays. Activation of PPARδ (A) and PPARγ (B) at the single concentration of the 5 selected ligands. B Fig. EC50 values of the compounds 1 and 2. C Fig. RMSD values for the protein backbone during the MD simulation. D Fig. RMSD values for the ligand atoms during the MD simulation. E Fig. Number of H-bonds between the selected ligands and the protein atoms during the MD simulation. A Table. 50 compounds selected by DOCK. These compounds were employed in the redocking analyses using GOLD and Surflex programs. (ZIP) [file pone.0118790.s001.zip › S1_File/B_Fig.tif]

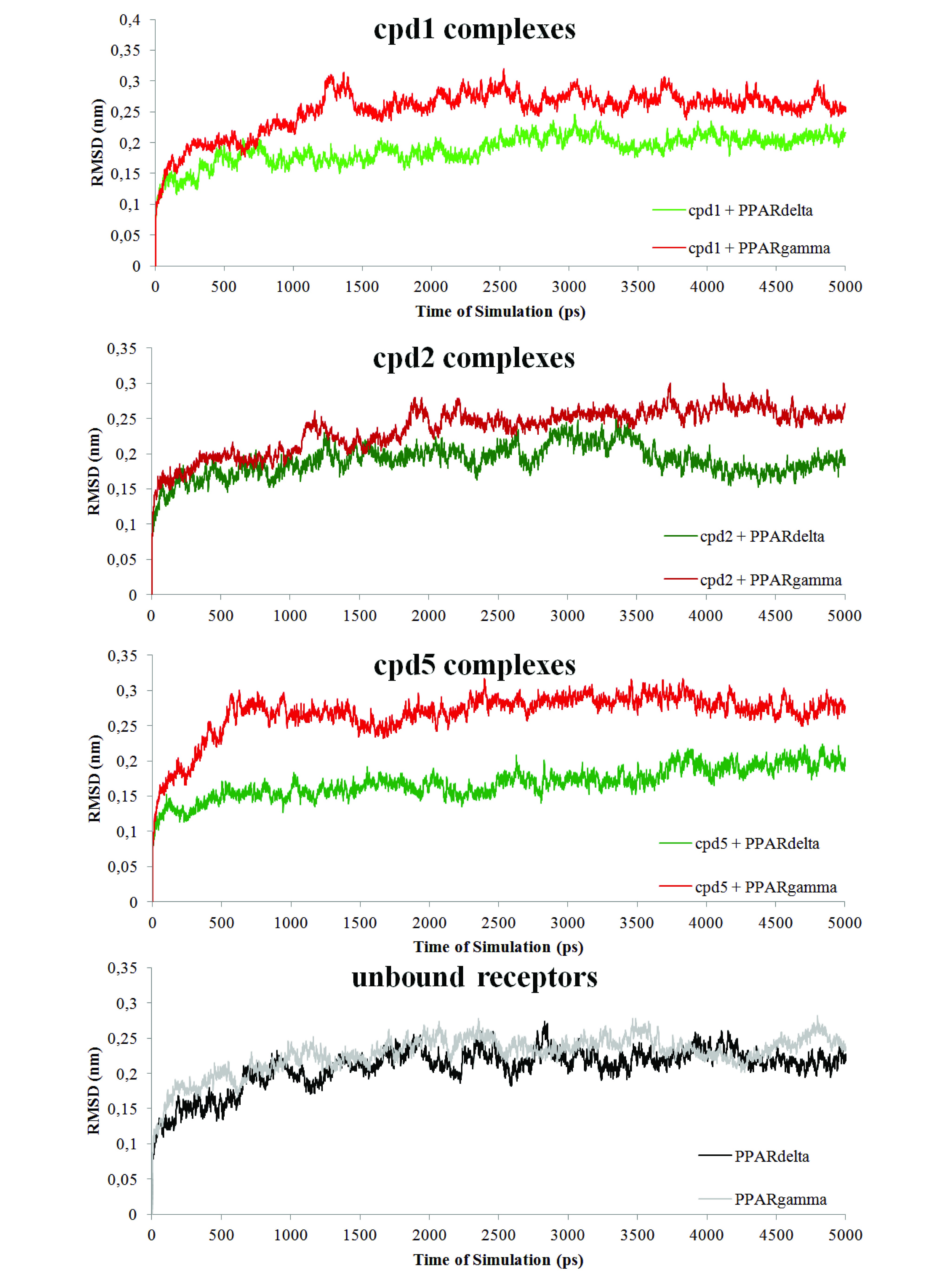

Supplement: S1 File — A Fig. Luciferase assays. Activation of PPARδ (A) and PPARγ (B) at the single concentration of the 5 selected ligands. B Fig. EC50 values of the compounds 1 and 2. C Fig. RMSD values for the protein backbone during the MD simulation. D Fig. RMSD values for the ligand atoms during the MD simulation. E Fig. Number of H-bonds between the selected ligands and the protein atoms during the MD simulation. A Table. 50 compounds selected by DOCK. These compounds were employed in the redocking analyses using GOLD and Surflex programs. (ZIP) [file pone.0118790.s001.zip › S1_File/C_Fig.tif]

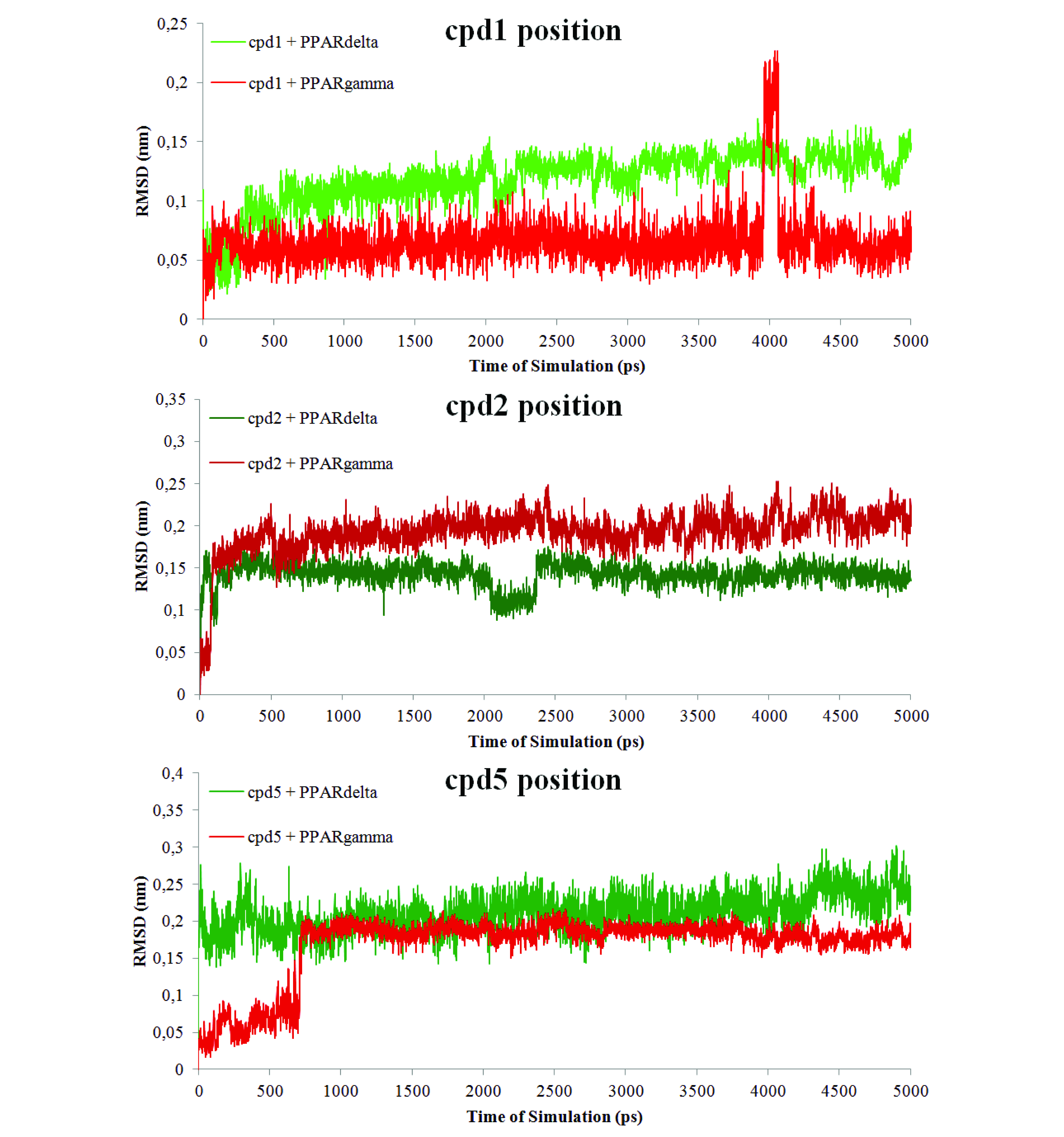

Supplement: S1 File — A Fig. Luciferase assays. Activation of PPARδ (A) and PPARγ (B) at the single concentration of the 5 selected ligands. B Fig. EC50 values of the compounds 1 and 2. C Fig. RMSD values for the protein backbone during the MD simulation. D Fig. RMSD values for the ligand atoms during the MD simulation. E Fig. Number of H-bonds between the selected ligands and the protein atoms during the MD simulation. A Table. 50 compounds selected by DOCK. These compounds were employed in the redocking analyses using GOLD and Surflex programs. (ZIP) [file pone.0118790.s001.zip › S1_File/D_Fig.tif]

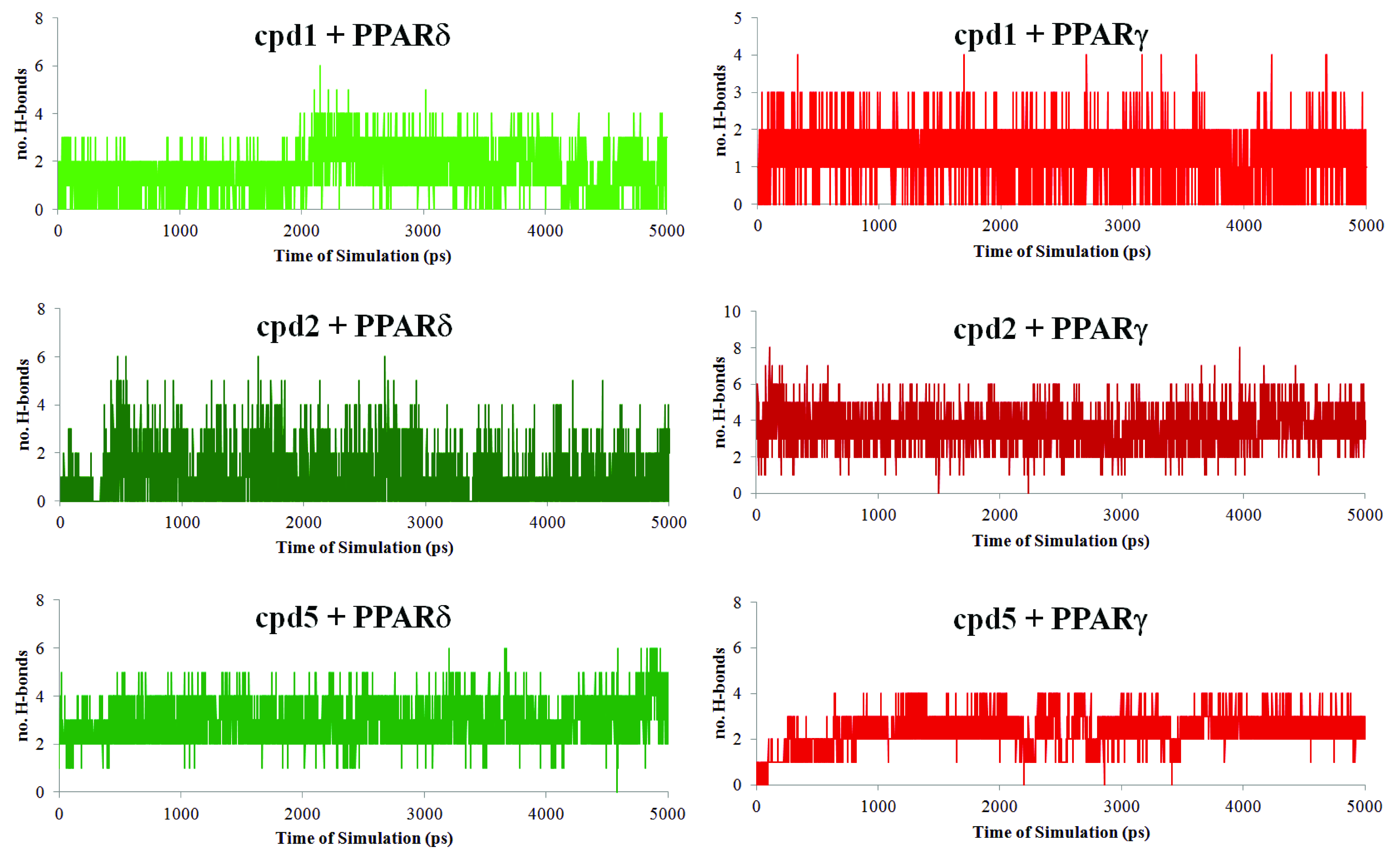

Supplement: S1 File — A Fig. Luciferase assays. Activation of PPARδ (A) and PPARγ (B) at the single concentration of the 5 selected ligands. B Fig. EC50 values of the compounds 1 and 2. C Fig. RMSD values for the protein backbone during the MD simulation. D Fig. RMSD values for the ligand atoms during the MD simulation. E Fig. Number of H-bonds between the selected ligands and the protein atoms during the MD simulation. A Table. 50 compounds selected by DOCK. These compounds were employed in the redocking analyses using GOLD and Surflex programs. (ZIP) [file pone.0118790.s001.zip › S1_File/E_Fig.tif]
